# Graded response model fit, measurement invariance and (comparative) precision of the Dutch-Flemish PROMIS® Upper Extremity V2.0 item bank in patients with upper extremity disorders

**DOI:** 10.1186/s12891-020-3178-8

**Published:** 2020-03-16

**Authors:** C. M. Lameijer, S. G. J. van Bruggen, E. J. A. Haan, D. F. P. Van Deurzen, K. Van der Elst, V. Stouten, A. J. Kaat, L. D. Roorda, C. B. Terwee

**Affiliations:** 1 Department of Trauma Surgery, Amsterdam UMC, Vrije Universiteit Amsterdam, De Boelelaan 1117, Amsterdam, 1081 HV the Netherlands; 2 Physical Therapy Sciences, Program in Clinical Health Sciences, University Medical Center Utrecht, Utrecht University, Utrecht, the Netherlands; 3 Department of Orthopedic Surgery, OLVG, Amsterdam, the Netherlands; 4 Department of Rheumatology, University Hospitals Leuven, Leuven, Belgium; 5 Department of Development and Regeneration, Skeletal Biology and Engineering Research Centre, KU Leuven - University of Leuven, Leuven, Belgium; 6 Department of Medical Social Sciences, Northwestern University, Feinberg School of Medicine, Chicago, IL USA; 7 Amsterdam Rehabilitation Research Center | Reade, Dr. Jan van Breemenstraat 2, Amsterdam, 1056 AB the Netherlands; 8 Department of Epidemiology and Biostatistics, Amsterdam Public Health Research Institute, Amsterdam UMC, Vrije Universiteit Amsterdam, Amsterdam, Netherlands

**Keywords:** Dutch-Flemish PROMIS, Upper extremity, Item response theory, Measurement invariance, Reliability

## Abstract

**Background:**

The Dutch-Flemish PROMIS® Upper Extremity (DF-PROMIS-UE) V2.0 item bank was recently developed using Item Response Theory (IRT). Unknown for this bank are: (1) if it is legitimate to calculate IRT-based scores for short forms and Computerized Adaptive Tests (CATs), which requires that the items meet the assumptions of and fit the IRT-model (Graded Response Model [GRM]);(2) if it is legitimate to compare (sub) groups of patients using this measure, which requires measurement invariance; and (3) the precision of the estimated patients’ scores for patients with different levels of functioning and compared to legacy measures. Aims were to evaluate (1) the assumptions of and fit to the GRM, (2) measurement invariance and (3) (comparative) precision of the DF-PROMIS-UE v2.0.

**Methods:**

Cross-sectional data were collected in Dutch patients with upper extremity disorders. Assessed were IRT-assumptions (unidimensionality [bi-factor analysis], local independence [residual correlations], monotonicity [coefficient H]), GRM item fit, measurement invariance (absence of Differential Item Functioning [DIF] due to age, gender, center, duration, and location of complaints) and precision (standard error of IRT-based scores across levels of functioning). To study measurement invariance for language [Dutch vs. English], additional US data were used. Legacy instruments were the Disability of the Arm, Shoulder and Hand (DASH), the QuickDASH and the Michigan Hand Questionnaire (MHQ).

**Results:**

In total 521 Dutch (mean age ± SD = 51 ± 17 years, 49% female) and 246 US patients (mean age ± SD = 48 ± 14 years, 69% female) participated. The DF-PROMIS-UE v2.0 item bank was sufficiently unidimensional (Omega-H = 0.80, Explained Common Variance = 0.68), had negligible local dependence (four out of 1035 correlations > 0.20), good monotonicity (H = 0.63), good GRM fit (no misfitting items) and demonstrated sufficient measurement invariance. Precise estimates (Standard Error < 3.2) were obtained for most patients (7-item short form, 88.5%; standard CAT, 91.3%; and, fixed 7-item CAT, 87.6%).

The DASH displayed better reliability than the DF-PROMIS-UE short form and standard CAT, the QuickDASH displayed comparable reliability. The MHQ-ADL displayed better reliability than the DF-PROMIS-UE short form and standard CAT for T-scores between 28 and 50. For patients with low function, the DF-PROMIS-UE measures performed better.

**Conclusions:**

The DF-PROMIS-UE v2.0 item bank showed sufficient psychometric properties in Dutch patients with UE disorders.

## Background

Upper extremity (UE) disorders impact health care, society and the lifes of patients. For instance in the field of orthopaedic and trauma surgery, UE disorders account for a large proportion of attendances to the Emergency Department with highest incidences in young patients and elderly females [1]. Total annual costs for all acute and chronic UE disorders are reported to be 290 million euro, of which wrist fractures are the most expensive injuries (83 million euro) due to high incidence, whereas upper arm fractures are most expensive per case (4440 euro) [1]. In addition, these disorders cause considerable losses in working days and productivity [2]. The disability caused by UE disorders significantly reduces physical, mental, and social health [2].

Patient-reported outcome measures (PROMs), consisting of validated questionnaires, are increasingly used in daily clinical practice to assess the impact of acute and chronic UE disorders on the lifes of patients. In the past, outcomes following these disorders were objectified using clinical measurements such as grip strength, range of motion, and radiological parameters. Nowadays the patient perspective on these outcomes is becoming more important. This may include the impact on physical health (e.g., physical functioning, pain intensity and interference), mental health (e.g., depression), and social health (e.g., ability to participate in social roles and activities).

The use of PROMs in daily clinical practice and for research purposes is not without problems. Many different PROMs have been developed and are being used in patients with UE disorders, including the Disability of the Arm, Shoulder and Hand (DASH) questionnaire [3], the QuickDASH [4], the Patient-Rated Wrist Evaluation (PRWE) [5], and the Michigan Hand Questionnaire (MHQ) [6]. Variation exists in their psychometric properties [7–10]. In addition, completing PROMs is time consuming for patients. Finally, the interpretation of the PROM scores is hampered by the variability of conditions the PROMs are applied to [8] and varies between them.

The Patient-Reported Outcomes Measurement Information System (PROMIS®) might offer a solution for some of the problems related to the use of traditional PROMs. The National Institutes of Health PROMIS® initiative has developed a new assessment system for measuring patient-reported health. The goal was to improve measurement quality and comparability of PROMs and reduce patients’ burden. Item banks were developed and validated for measuring specific symptoms and health status domains [11, 12]. An item bank is a universal (non-disease specific) applicable set of items (questions) with responses (answers) that all measure the same domain (construct or concept) [13]. The items of a bank are calibrated on a scale, using a modern psychometric technique, called Item Response Theory (IRT) modelling. In this way, people and items are located on the same scale (ruler or metric) according to their “difficulty”. For PROMIS, the score is expressed as a T-score, which is a standardized score, with 50 currently representing the average score of the US general population, with a standard deviation of 10. IRT-based item banks enable the use of short forms (fixed subsets of items from the item bank) and Computerized Adaptive Testing (CAT). CAT uses an algorithm that selects the most informative items from the item bank, based on the individual’s response to previously administered items. In this way, high measurement precision can be obtained with low respondent burden [11, 14].

PROMIS included an item bank that measures UE-related physical functioning and this bank has recently been updated, from v1.2 to v2.0, to measure a wider range of upper extremity functioning and showed higher precision when used in patients with UE disorders [15]. The v2.0 item bank was translated into Dutch-Flemish (DF-PROMIS-UE v2.0) and some of the psychometric properties of this bank have been studied in patients with UE disorders from a general [16] and an academic hospital [17]. Evidence was found for the following psychometric properties: internal consistency [17], structural validity [17], construct validity [16, 17] and cross-cultural validity [16]. In addition, absence of floor and ceiling effects in the full bank and the 7-item short form was shown [16].

Some other important psychometric properties of the DF-PROMIS-UE v2.0 item bank still need to be evaluated. Unknown for the DF-PROMIS-UE v2.0 bank are: (1) if it is legitimate to calculate IRT-based scores for short forms and Computerizes Adaptive Tests (CATs), which requires that the items meet the assumptions of and fit to the IRT-model (in this case the Graded Response Model [GRM]);(2) if it is legitimate to compare (sub) groups of patients using the measure at issue, which requires measurement invariance; and (3) the precision of the estimated patients’ scores for patients with different levels of functioning and compared to legacy measures. Therefore, the aims of this study were to evaluate (1) the assumptions of and fit to the GRM, (2) measurement invariance and (3) (comparative) precision of the DF-PROMIS-UE v2.0 item bank in patients with UE disorders in comparison to legacy instruments Disability of Arm Shoulder and Hand (DASH) questionnaire, QuickDASH and Michigan Hand Questionnaire (MHQ).

## Methods

### Participants

Patients visiting the outpatient department of trauma surgery at a level 1 traumacenter or the outpatient department of orthopaedic surgery at a level 2 traumacenter, between February 2018 and August 2018, were invited to participate. Patients were eligible if they were 18 years or older, had an UE disorder, were able to read Dutch and provided informed consent. Because we deemed a sample of at least 500 patients mandatory for item parameter estimation, data of studies performed by van Bruggen et al. [17] and Haan et al. [16] were pooled [18]. To study measurement invariance for language, we used additional data of US patients from an online panel, aged 18 years or older, who endorsed having some difficulty due to UE pain or function [15].

### Measures

Besides demographic and disease specific questions, the questionnaire included the full DF-PROMIS-UE v2.0 item bank. In addition, the questionnaire contained 3 disease-specific legacy instruments: the DASH, the QuickDASH and the MHQ (Table 1).

**Table 1:** Legacy instruments

DASH	30 items (addressed to disabilities and symptoms in musculoskeletal disorders of the upper limbs). Timeframe: during the last week.
	Six different 5-point Likert response scales: • No difficulty/Mild difficulty/Moderate difficulty/Severe difficulty/Unable • Not at all/Slightly/Moderately/Quite a bit/Extremely • Not limited at all/Slightly limited/Moderately limited/Very limited/Unable • None/Mild/Moderate/Severe/Extreme • No difficulty/Mild difficulty/Moderate difficulty/Severe difficulty/So much difficulty that I can’t sleep • Strongly disagree/Disagree/Neither agree or disagree/Agree/Strongly agree.
	Higher scores imply more disability: 0 (no disability) to 100 (most severe disability).
QuickDASH	11 items (addressed to disabilities and symptoms in musculoskeletal disorders of the upper limbs). Timeframe: during the last week.
	Two different 11-point response scales: • Pain: 0 (no pain) to 10 (unbearable pain) • Function: 0 (no disability) to 10 (most disability)
	Higher scores imply more disability: 0 (no disability) to 100 (most severe disability).
MHQ-ADL	7 items (addressed to activities of daily living). Timeframe: during the last week.
	One 5-point Likert response scale: • Not difficult at all/A little difficult/Somewhat difficult/Moderately difficult/Very difficult.
	Higher scores imply less disability: 0 (Very difficult to do) to 100 (not difficult to do at all).

Abbreviations in alphabetic order: *DASH* Disability of Arm, Shoulder and Hand, *MHQ-ADL* Michigan Hand Questionnaire-Activities of Daily Living subscale

The DF-PROMIS-UE v2.0 item bank contains 46 items addressing upper extremity function. There are two different 5-point Likert response scales: 1) Unable to do/With much difficulty/With some difficulty/With a little difficulty/Without any difficulty; 2) Cannot do/Quite a lot/Somewhat/Very little/Not at all. There is no timeframe for the items, but current status is inferred. Higher scores indicate better function. A 7-item short form was developed. In addition, the item bank can be used as CAT. The total score of the DF-PROMIS-UE v2.0 item bank, short form or CAT is not a sum or total score, but a weighted score, based on the underlying IRT-model, taking the difficulty of the items into account. All scores are expressed as a T-score, which is a standardized score, with 50 currently representing the average score of the US general population, with a standard deviation of 10, and higher scores indicate more of the domain at issue, in this case better UE-related physical functioning.

The DASH questionnaire contains 30 items, specifically addressed to physical function and symptoms in musculoskeletal disorders of the upper extremity (Table 1) [3]. Both the original English DASH and the official Dutch translation were found to have sufficient psychometric properties [19–21].

The QuickDASH is an 11-item shortened version of the DASH (Table 1). Using conceptual methods these 11 items were selected from the total DASH questionnaire based on the criteria: 1) number of items with > 40% in one response category, 2) Cronbach’s alpha > 0.90 and 3) highest correlation with the 30-item DASH and with other markers of physical function and severity of problem. The QuickDASH has sufficient psychometric properties [4].

The MHQ is a hand-specific instrument that measures several domains and is applicable to patients with conditions of, or injury to, the hand and wrist (Table 1) [6]. The MHQ contains six distinct subscales. In this study, we used the MHQ subscale Activities of Daily Living (MHQ-ADL), which assesses difficulty in performing daily activities for the right hand (5 items), for the left hand (5 items) and both hands (7 items). We used the 7 items referring to both hands because this corresponds most with the generic PROMIS items. The psychometric properties of the MHQ score were found to be sufficient [6, 22–26].

### Procedures

The study was approved by the local medical ethics committees of the participating hospitals. Consenting patients were requested to complete all 46 items of the DF-PROMIS-UE v2.0 item bank through an online survey or, only if preferred, using a paper version of the questionnaire. In addition, patients completed general questions regarding age, gender, education and ethnicity. Also questions regarding type of injury and duration of complaints were included. In addition, the DASH, which encompasses the QuickDASH, and the MHQ were completed.

### Statistical analysis

#### IRT-model assumptions and fit

The psychometric analyses were conducted using the original PROMIS analysis plan [14]. For an item bank it is important to know if it is legitimate to calculate IRT-based scores for short forms and CATs. This requires, firstly, that the items meet the assumptions of an IRT-model and, secondly, fit to the IRT-model at issue. An IRT-model requires that the following four assumptions are met: unidimensionality, local independence, monotonicity and measurement invariance [14, 27].

Studying the first IRT-assumption, unidimensionality, addresses the research question whether the items assessed one construct, in this case UE-related physical function. Unidimensionality was evaluated using multiple methods:

- Confirmatory Factor Analyses (CFA). The CFA was conducted on the polychoric correlation matrix with Weighted Least Squares with Mean and Variance adjustment (WLSMV) estimation, using the R package LAVAAN (version 0.5–23.1097) [28]. Fit of the unidimensional model was evaluated using the following parameters: Chi-square, df, *p-*value, Comparative Fit Index (CFI), Tucker-Lewis Index (TLI), Root Means Square Error of Approximation (RMSEA) with 90% CI and Standardized Root Mean Residual (SRMR) [28]. We reported scaled fit indices, which are considered more exact than unscaled indices. Sufficient evidence for unidimensionality and thus adequate model fit was considered if CFI > 0.95, TLI > 0.95, RMSEA < 0.06 and a SRMR < 0.08 [14, 27, 29].
- Exploratory Factor Analysis (EFA). EFA was carried out on the polychoric correlation matrix with WLSMV estimation procedures using the R package Psych (version 1.7.5) [18]. Unidimensionality was considered sufficient when the first factor accounts for at least 20% of the variability and when the ratio of the variance explained by the first to the second factor is greater than 4 [14].
- Exploratory bi-factor analysis was performed when CFA showed a poor model fit. Bi-factor analysis evaluates, when multidimensionality is present, the impact of multidimensionality. Exploratory bi-factor analysis was conducted using the R package Psych (version 1.7.5). Criteria were: omega H and Explained Common Variance (ECV). Coefficient omega H > 0.80 [30] and ECV > 0.60 [31] indicates that the risk of biased parameters, when fitting multidimensionality data into a unidimensional model, is low.
- When suspicion for lack of unidimensionality was present, an additional forced two-factor analysis EFA with Varimax rotation was performed in SPSS (version 26).

Evaluating the second IRT-assumption, local independence, addresses the research question whether the items are only related to the construct (the dominant factor) being measured and not to other constructs (any other factors). This implies that, after controlling for the dominant factor, there should be no significant covariance between item responses. Local independence was evaluated by examining the residual correlation matrix resulting from the single factor CFA. A value of 0.20 above the average residual correlation was taken as a critical value for local dependence [32].

Studying the third IRT-assumption, monotonicity, addresses the research question whether the probability of an affirmative response to the items increases with increasing levels of the underlying construct. This implies, e.g., in case the item responses “Unable to do/With much difficulty/With some difficulty/With a little difficulty/Without any difficulty”, that the probability of endorsing a higher item response category, e.g., choosing “Without any difficulty” instead of “With a little difficulty”, should increase with increasing levels of the underlying construct, in this case the UE-related physical functioning. Monotonicity was evaluated by fitting a non-parametric IRT model, using Mokken scaling in the R package Mokken (version 2.8.4) [33, 34]. We evaluated the fit of the model by calculating the scalability coefficient H per item and for the total scale. We considered monotonicity acceptable if the scalability coefficients for the items were ≥ 0.30 and for the total scale ≥0.50 [33].

Evaluating the fourth IRT-assumption, measurement invariance, addresses the research question whether it is legitimate to compare (sub) groups of patients using the measure at issue. Item parameters should be equivalent between (sub) groups, e.g., age or gender groups, implying that there should be absence of Differential Item Functioning (DIF)*.* DIF analyses are used to examine if people from different (sub) groups, e.g. males versus females, with the same level of the construct, e.g. the same level of UE-related physical functioning, have different probabilities of giving a certain response to an item [14, 35, 36]. Uniform DIF exists when the DIF is consistent, with the same magnitude of DIF across the entire range of the construct [14, 35, 36]. In this case the item location parameters differ between the (sub)groups. Non-uniform DIF exists when the magnitude or direction of DIF differs across the construct. In this case the item discrimination parameters differ between the (sub)groups. DIF was evaluated with use of the R package Lordif (version 0.3–3), using ordinal logistic regression models with a McFadden’s pseudo *R*^2^ change of 2% as critical value [14, 37, 38]. DIF was evaluated for age (median split: < 53 years versus ≥53 years), gender, duration of complaints (< 6 months versus ≥6 months), center (level 1 versus level 2 traumacenter) and primary location of complaints (hand/wrist versus arm/shoulder). Regarding location of complaints, patients were able to report on multiple areas. For the DIF analysis regarding location of complaints we used patients who reported either pain in shoulder/arm or hand/wrist only. Measurement invariance for language is a key aspect of cross-cultural validity and was addressed by a DIF analysis for language (Dutch-Flemish versus American-English). In the US dataset some response categories had insufficient responses for analysis and these categories had to be collapsed. In order to compare our population with the US population, scores on the response categories “without much difficulty” and “unable to do” were therefore collapsed for 8 items (PFA43r1, PFB16r1, PFB19r1, PFB20r1, PFB21r1, PFB23r1, PFB31r1, and PFB37r1). For item PFB15r1 the response categories ‘with some difficulty’, ‘without much difficulty’ and ‘unable to do’ were collapsed, according to the US PROMIS convention [39]. The impact of DIF on total scores was examined by plotting the differences between the initial theta and theta corrected for DIF.

After evaluation of the IRT-assumptions, the IRT-model at issue, in this case the logistic Graded Response Model (GRM) which is an IRT-model for ordinal data, was fit to the item response data. The GRM model yields two item types of parameter estimates: the item thresholds and the item slope [35]. Item threshold parameters locate item response categories along the scale (i.e. the construct of interest) [35]. The item slope parameter refers to the discriminative ability of the items, with higher slope values indicating a stronger relationship to the construct of interest [35]. For items with five response categories, four item thresholds were estimated. To assess the fit of the GRM we used the R-package Mirt (version 3.3.2) [40]. To assess the degree to which possible misfit affects the IRT-model, a generalization of Orlando and Thissen’s S-X^2^ for polytomous data was used [41]. These statistics compare the observed and expected response frequencies under the estimated IRT model and quantifies the differences between the observed and expected response frequencies. Items with a S-X^2^*p-*value ≤0.001 demonstrate poor fit [14, 42].

#### Precision

Measurement precision (reliability) is conceptualized within IRT as “‘information”. In the context of IRT the measurement precision can differ across levels of the measured construct (*θ* = Theta). The relationship between information (I) and standard error (SE) is defined by the formula.

SE (θ) = 1/√*I*(θ), where SE is the standard error of the estimated θ, *I* is information and θ is the estimated level of the construct. For each patient, we calculated four T-scores: one based on all items of the item bank, one based on the standard 7-item short form, and two based on CAT simulations. In the first simulated CAT we used the standard PROMIS CAT stopping rules. The standard CAT stops if a SE of 3 on the T-score metric is reached, comparable to a reliability slightly higher than 0.90, or a maximum of 12 items has been administered. The recommended minimum of four items was not used because this could not be specified in the R-package at issue. In the second simulated CAT we administered a fixed number of seven items to compare the reliability of this CAT with the 7-item short form. In all simulations the starting item was the item with the highest information value for the average level of functioning in our study population (theta = 0) (http://www.healthmeasures.net/score-and-interpret/calculate-scores). All PROMIS T-scores were calculated using the US item parameters (http://www.healthmeasures.net/score-and-interpret/calculate-scores). We used the R-package catR (version 3.12) and *expected* a posteriori (EAP) estimations for the CAT simulations [18]. The SEs across T-scores for the entire item banks were plotted, for the standard 7-item short form, and for the two different CAT simulations. In addition, the distribution of T-scores in our population was plotted. This enabled us to relate the reliability of the item bank to the distribution of T-scores in this population.

To compare the precision of the DF-PROMIS-UE v2.0 item bank to the precision of the DASH, QuickDASH and the MHQ-ADL (comparative precision), we also fitted a GRM on these three legacy instruments. The scoring of the DASH and QuickDASH was reversed resulting in higher scores indicated better functioning, comparable to PROMIS. We plotted the Standard Errors (SEs) of the T-scores of the DASH, QuickDASH and MHQ-ADL in addition to the SEs of the T-scores of the DF-PROMIS-UE v2.0 short form and standard CAT.

In addition, relative efficiency was quantified per patient for each measure as Information ((1/SE)^2^) divided by the number of items administered. Relative efficiency among the instruments was calculated as the mean efficiency of the PROMIS measures divided by the mean efficiency of the legacy measures. If the mean relative efficiency is larger than 1, the PROMIS measure is on average more efficient (more information per item) than the legacy instrument, but if it is less than 1, the legacy instrument is on average more efficient.

## Results

Of the 828 invited eligible patients, 624 (75%) (405 of 524 level 1 center and 218 of 304 level 2 center) provided informed consent. Of these 624 consenting patients, 103 (all level 1) did not complete the questionnaire, even after two reminders by email. Of the remaining 521 (303 level 1 center and 218 level 2 center, total response rate 63%) patients, 515 fully completed the DF-PROMIS-UE v2.0 item bank. Most analyses were performed on 521 patients. The CAT simulations were performed on the 515 cases with complete DF-PROMIS-UE response data. The DIF analyses for location of complaints were based on 337 patients (268 patients who reported complaints in shoulder/arm only and 68 patients who reported complaints in the hand/wrist only).

### Demographic and clinical characteristics

Demographic and clinical characteristics of the Dutch and US samples are summarized in Table 2. The mean age of the Dutch population was 51 years (SD 17) and 253 (49%) were female.

**Table 2:** Demographic and clinical characteristics of the Dutch and US samples

	Dutch sample (*n* = 521)			US sample (*n* = 246)
	Level 1 center (*n* = 303)	Level 2 center (*n* = 218)	Total (*n* = 521)	
Age, mean (SD)	50 (17)	53 (15)	51 (17)	48 (14)
Gender, n (%)				
Male	159 (53)	109 (50)	268 (51)	76 (31)
Female	144 (47)	109 (50)	253 (49)	170 (69)
Country of birth, n (%)				
Netherlands	276 (91)	161 (65)	437 (86)	
Other	27 (9)	44 (20)	71 (14)	
Missing	0 (0)	13 (15)	0 (0)	
Social status, n (%)				
Single	110 (36)	69 (32)	179 (34)	
Married/living together	155 (51)	127 (58)	282 (54)	
Living apart together	15 (5)	4 (2)	19 (4)	
Living with parents	16 (5)	6 (3)	22 (4)	
Other	7 (3)	12 (5)	19 (4)	
Educational level, n (%)				
< high school degree	34 (11)	40 (18)	74 (15)	6 (2)
High school degree	99 (33)	75 (33)	174 (33)	53 (22)
Some college	16 (5)	14 (6)	30 (6)	81 (33)
College degree	122 (40)	72 (33)	194 (37)	80 (32)
Advanced degree	32 (11)	17 (8)	49 (9)	26 (11)
Employment status, n (%)				
Full time	141 (47)	84 (39)	217 (43)	
Part time	55 (18)	40 (18)	93 (18)	
Student	20 (7)	5 (2)	25 (5)	
Unpaid/volunteer/household	13 (4)	18 (8)	31 (6)	
Retired	49 (16)	40 (18)	88 (17)	
Unemployed	6 (2)	10 (5)	14 (3)	
Other	19 (6)	21 (10)	40 (8)	
Duration of complaints, n (%)				
< 1 month	135 (45)	22 (10)	157 (30)	
1–3 months	39 (13)	22 (10)	61 (12)	
3–6 months	42 (14)	30 (14)	72 (14)	
*< 6 months (DIF)*	*216 (72)*	*74 (34)*	*290 (56)*	
6–12 months	20 (7)	36 (17)	56 (11)	
1–2 years	8 (3)	46 (21)	54 (10)	
2–5 years	2 (1)	31 (14)	33 (6)	
5 years	1 (0)	31 (14)	32 (6)	
*≥ 6 months (DIF)*	*31 (11)*	*144 (66)*	*175 (33)*	
Unknown/missing	56 (19)	0 (0)	56 (11)	
Location of pain^a^, n (%)				
Shoulder(s)	137 (45)	190 (87)	318 (63)	
Arm(s)	125 (41)	142 (65)	259 (51)	
*Shoulder/arm (DIF)*^b^	*132 (44)*	*136 (62)*	*268 (80)*	
Hand(s)	105 (35)	59 (27)	161 (32)	
Finger(s)	64 (21)	49 (22)	112 (22)	
*Hand/wrist (DIF)*^b^	*62 (21)*	*7 (3)*	*69 (20)*	
DF-PROMIS-UE v2.0, mean (SD) T-scores	34.7 (3.6)	33.4 (9.1)	33.9 (8.9)	36.5 (7.0)
DASH, mean (SD) T-scores	35.6 (22.1)	36.5 (21.0)	35.9 (21.6)	
QuickDASH, mean (SD) T-scores	36.8 (22.1)	38.1 (21.8)	37.3 (22.0)	
MHQ-ADL, mean (SD) T-scores	61.4 (31.0)	74.5 (25.6)	66.7 (29.6)	

Abbreviations in alphabetic order: *DASH* Disability of Arm, Shoulder and Hand, *DF-PROMIS-UE v2.0* Dutch-Flemish translated version of the PROMIS Upper Extremity v2.0 item bank, *DIF* Differential item functioning, *MHQ-ADL* Michigan Hand Questionnaire-Activities of Daily Living subscale, *n* Number, *SD* Standard deviation, *%* Percentage

^a^Multiple answers were allowed, ^b^For the DIF analysis regarding location of complaints only patients who reported either pain in shoulder/arm or hand/wrist were included

### IRT-model assumptions and fit

The results of the psychometric analyses are summarized in Tables 3 and 4.

**Table 3:** Results with respect to the IRT-model assumptions of the DF-PROMIS-UE v2.0 bank

Analyses	Outcome	Result
**IRT assumptions and model fit**		
Confirmatory Factor Analysis of one-factor model	Chi square	5964.333
	df	989
	*p*-value	0.000
	Scaled CFI	0.93
	Scaled TLI	0.93
	Scaled RMSEA (90% CI)	0.099 (0.097–0.101)
	Scaled SRMR	0.09
Exploratory Factor Analysis	Eigenvalue first factor	30.1
	Eigenvalue second factor	2.8
	Ratio	10.7
Exploratory bi-factor analysis	ECV	0.68
	Omega-H	0.80
Local Dependency	Residual correlation > 0.17	4 item pairs locally dependent (3.3%)
Monotonicity	Scalability coefficient H	0.63
	Scalability coefficients H_i_	Range 0.55–0.70

Abbreviations in alphabetic order: *CFI* Comparative Fit Index, *ECV* Explained Common Variance, *RMSEA* Root Means Square Error of Approximation, *SRMR* Standardized root mean residual, *TLI* Tucker-Lewis Index

**Table 4:** Result with respect to the monotonicity assumption and GRM-model fit at the item level, GRM-model item parameters, and measurement invariance of the DF-PROMIS-UE v2.0 bank

Item		Monotonicity	GRM-model fit	GRM-model Item parameters					Measurement invariance									
ID	Item stem	Scalability coefficient H_i_	S-X^2^ *p*-value	a	b1	b2	b3	b4	Gender		Center		Duration of complaints		Location of complaints		Language	
									UF	*R*^2^	UF	*R*^2^	UF	*R*^2^	UF	*R*^2^	UF	*R*^2^
PFA14r1^ab^	Are you able to carry a heavy object (over 10 pounds/5 kg)?	0.591	0.181	1.862	−0.613	−0.227	0.415	1.054										
PFA16r1	Are you able to dress yourself, including tying shoelaces and buttoning your clothes?	0.639	0.021	2.780	−1.757	−1.097	− 0.397	0.444										
PFA17	Are you able to reach into a high cupboard?	0.550	0.677	1.670	− 1.156	−0.587	−0.046	0.657							UD	0.118		
PFA18	Are you able to use a hammer to pound a nail?	0.656	0.451	3.027	−0.768	−0.421	− 0.059	0.361										
PFA20	Are you able to cut your food using eating utensils?	0.642	0.003	3.028	−1.553	−0.985	−0.376	0.172			UD	0.032						
PFA28	Are you able to open a can with a hand can opener?	0.679	0.063	3.404	−0.738	−0.431	− 0.111	0.463			UD	0.027						
PFA29r1	Are you able to pull heavy objects (10 pounds/5 kg) towards yourself?	0.629	0.363	2.266	−1.068	−0.534	−0.066	0.771										
PFA34^ab^	Are you able to wash your back?	0.604	0.353	1.989	−0.906	− 0.343	0.249	1.077							UD	0.028		
PFA35	Are you able to open and close a zipper?	0.606	0.247	2.572	−2.203	−1.347	−0.704	0.099										
PFA36^ab^	Are you able to put on and take off a coat or jacket?	0.579	0.408	1.968	−2.736	−1.438	−0.495	0.604							UD	0.043		
PFA38	Are you able to dry your back with a towel?	0.622	0.596	2.378	−1.488	−0.923	−0.181	0.682							UD	0.028		
PFA40	Are you able to turn a key in a lock?	0.611	0.729	2.545	−1.992	−1.446	−0.922	−0.342										
PFA43r1	Are you able to write with a pen or pencil?	0.592	0.242	2.345	−1.857	−1.371	−0.843	−0.401										
PFA44	Are you able to put on a shirt or blouse?	0.606	0.4822	2.222	−2.361	−1.439	−0.624	0.473										
PFA48	Are you able to peel fruit?	0.634	0.149	2.983	−1.187	−0.877	− 0.491	0.058			UD	0.050			UD	0.027		
PFA50	Are you able to brush your teeth?	0.612	0.211	2.310	−2.416	−2.001	−1.292	−0.614										
PFA54	Are you able to button your shirt?	0.630	0.140	2.783	−1.871	−1.330	−0.730	0.084			UD	0.023	UD	0.020	UD	0.023		
PFB11	Are you able to wash dishes, pots, and utensils by hand while standing at a sink?	0.638	0.251	2.928	−1.421	−0.825	−0.375	0.303										
PFB13^ab^	Are you able to carry a shopping bag or briefcase?	0.593	0.010	1.926	−1.262	−0.808	−0.126	0.580							UD	0.027		
PFB15r1	Are you able to change the bulb in a table lamp?	0.641	0.768	3.067	−1.280	−1.012	−0.571	−0.001										
PFB16r1	Are you able to press with your index finger (for example ringing a doorbell)?	0.596	0.071	2.165	−2.651	−2.110	−1.510	−0.950									UD	0.025
PFB18	Are you able to shave your face or apply makeup?	0.645	0.377	3.073	−1.810	− 1.411	− 0.841	− 0.101										
PFB19r1	Are you able to squeeze a new tube of toothpaste?	0.663	0.518	3.177	−2.142	−1.667	−1.055	−0.410										
PFB20r1	Are you able to cut a piece of paper with scissors?	0.651	0.071	3.313	−1.624	−1.229	−0.844	− 0.310							UD	0.021		
PFB21r1	Are you able to pick up coins from a table top?	0.603	0.013	2.164	−2.350	−1.916	−1.389	−0.677									UD	0.043
PFB22	Are you able to hold a plate full of food?	0.662	0.489	2.965	−1.488	− 1.153	−0.639	0.103										
PFB23r1^b^	Are you able to poor liquid from a bottle into a glass?	0.661	0.002	3.046	−1.692	−1.373	−0.798	−0.151										
PFB25	Are you able to push open a door after turning the knob?	0.593	0.257	2.157	−2.353	−1.524	−0.966	− 0.182										
PFB26	Are you able to shampoo your hair?	0.644	0.331	2.907	−1.470	−1.009	− 0.496	0.287										
PFB27	Are you able to tie a knot or a bow?	0.640	0.015	3.027	−1.429	−0.959	−0.570	0.042			UD	0.056	UD	0.031				
PFB28r1^a^	Are you able to lift 10 pounds (5 kg) above your shoulder?	0.639	0.081	2.040	−0.198	0.224	0.670	1.350							UD	0.062		
PFB30	Are you able to open a new milk carton?	0.675	0.035	3.590	−1.449	−0.990	−0.520	0.100										
PFB31r1	Are you able to open car doors?	0.654	0.181	2.906	−1.773	−1.330	−0.798	−0.181										
PFB33	Are you able to remove something from your back pocket?	0.577	0.478	2.045	−1.626	−1.104	−0.576	0.209										
PFB34^ab^	Are you able to change a light bulb overhead?	0.638	0.311	2.357	−0.717	−0.357	0.079	0.824							UD	0.052		
PFB36	Are you able to put on a pullover sweater?	0.595	0.475	2.061	−2.009	−1.148	−0.265	0.618							UD	0.058		
PFB37r1	Are you able to reach and get down a 5 pound (2 kg) object from above your head?	0.660	0.724	3.125	−1.980	− 1.547	−0.978	0.348										
PFB39r1	Are you able to reach and get down a 5 pound (2 kg) object from above your head?	0.626	0.605	2.218	−0.886	−0.533	− 0.055	0.705					UD	0.022	UD	0.030		
PFB41	Are you able to trim your fingernails?	0.586	0.595	2.352	−1.487	−1.091	−0.547	0.001			UD	0.038						
PFB56r1	Are you able to lift one pound (0.5 kg) to shoulder level without bending your elbow?	0.563	0.250	1.816	−1.004	−0.604	−0.191	0.508							UD	0.041		
PFC43	Are you able to use your hands, suchs as for turning faucets, using kitchen gadgets, or sewing?	0.619	0.045	2.853	−1.755	−1.243	−0.713	−0.069			UD	0.232			UD	0.024		
PFC49	Are you able to water a house plant?	0.662	0.016	3.091	−1.807	−1.431	−1.056	−0.463	UD	0.028							UD	0.022
PFM2^b^	Are you able to lift a heavy painting or picture to hang on your wall above eye-level?	0.684	0.720	2.786	−0.431	−0.083	0.377	1.208									NUD	0.021
PFM16^ab^	Are you able to pass a 20-pound (10 kg) turkey or ham to other people at the table?	0.697	0.275	2.698	−0.212	0.176	0.677	1.469										
PFM18^b^	Are you able to continuously swing a baseball bat or tennis racket back and forth for 5 min?	0.624	0.131	1.941	−0.339	0.091	0.553	1.239							UD	0.028		
PFC8	Does your health now limit you in opening a previously opened jar?	0.617	0.203	2.460	−1.903	0.999	−0.365	0.391										

Abbreviations in alphabetical order: *ID* Identification, *GRM* Graded Response Model, *NUD* Non-Uniform DIF, *ID*, *UD* Uniform DIF, *UF* Uniformity

^a^ items included in the 7a short form

^b^ items selected as one of the first three items in the CAT

#### Unidimensionality

The results indicated unidimensionality, although not all criteria for unidimensionality were met (Table 3). The CFA results showed some lack of unidimensionality. The EFA and the bi-factor analysis supported unidimensionality. The forced two-factor analyses showed some evidence for a 2-factor model, including one factor consisting of items referring to using the shoulder or lifting heavy objects (eigenvalue 26.1) and one factor consisting of items referring to fine tactile function (eigenvalue 3.3) (Appendix 1).

#### Local dependence

Thirty-seven percent of the residual correlations were positive. The average residual correlation was − 0.033, so the critical value 0.20 above the mean would be 0.17 [32]. Four residual correlations (out of 1035 correlations, 0.004%) were larger than 0.17, suggesting local dependence: PFA14r1 (‘Are you able to carry a heavy object (over 10 pounds /5 kg)?’) had a residual correlation of 0.214 with PFA29r1 (‘Are you able to pull heavy objects (10 pounds/ 5 kg) towards yourself?’), PFA36 (‘Are you able to put on and take off a coat or jacket?’) had a residual correlation of 0.221 with PFA44 (‘Are you able to put on a shirt or blouse?’), a residual correlation of 0.184 with PFB36 (‘Are you able to put on a pullover sweater?’) and a residual correlation of 0.173 with PFA34 (‘Are you able to wash your back?’) respectively. An additional 32 item pairs had negative residual correlations > − 0.20, suggesting multidimensionality.

#### Monotonicity

The scalability coefficients H_i_ of the items ranged from 0.55 (PFA17 *‘Are you able to reach into a cupboard?’*) to 0.70 (PFM16 ‘*Are you able to pass a 20-pound (10kg) turkey or ham to other people at the table?’*) for the individual items (Table 4). The Mokken scalability coefficient H for the entire item bank was 0.63. Therefore, the DF-PROMIS-UE v2.0 items sufficiently met the monotonicity assumption.

#### Measurement invariance

No DIF was found for age, one item was flagged for DIF regarding gender, 7 items were flagged for DIF regarding center, three items were flagged for DIF regarding duration of complaints, and 15 items were flagged for DIF regarding location of complaints (Table 4). The combined impact of the DIF items on total scores was negligible for all DIF variables (as an example, Appendix 2 shows the differences between the initial theta and theta corrected for DIF for location of complaints; 75% of these differences were roughly between − 0.075 and 0.06 theta points). When analyzing DIF for language, one item was flagged for non-uniform DIF and three items were flagged for uniform DIF (Table 4). The impact of DIF for language on the total score was negligible providing evidence for cross-cultural validity (Table 4).

#### GRM fit

There were no misfitting items (Table 4). On the Dutch metric, the item thresholds ranged from − 2.7 (PFA36 ‘*Are you able to put on and take off a coat or jacket?’)* to 1.5 (PFM16 ‘*Are you able to pass a 20-pound (10kg) turkey or ham to other people at the table?’)* (min/max of all thresholds)*.* The item discrimination parameters ranged from 1.7 to 3.6. The item with lowest discriminative ability was PFA17 (*‘Are you able to reach into a cupboard?’*) and PFB30 (*‘Are you able to open a new milk carton?’)* was the item with highest discriminative ability.

### Precision

**Fig. 1:**
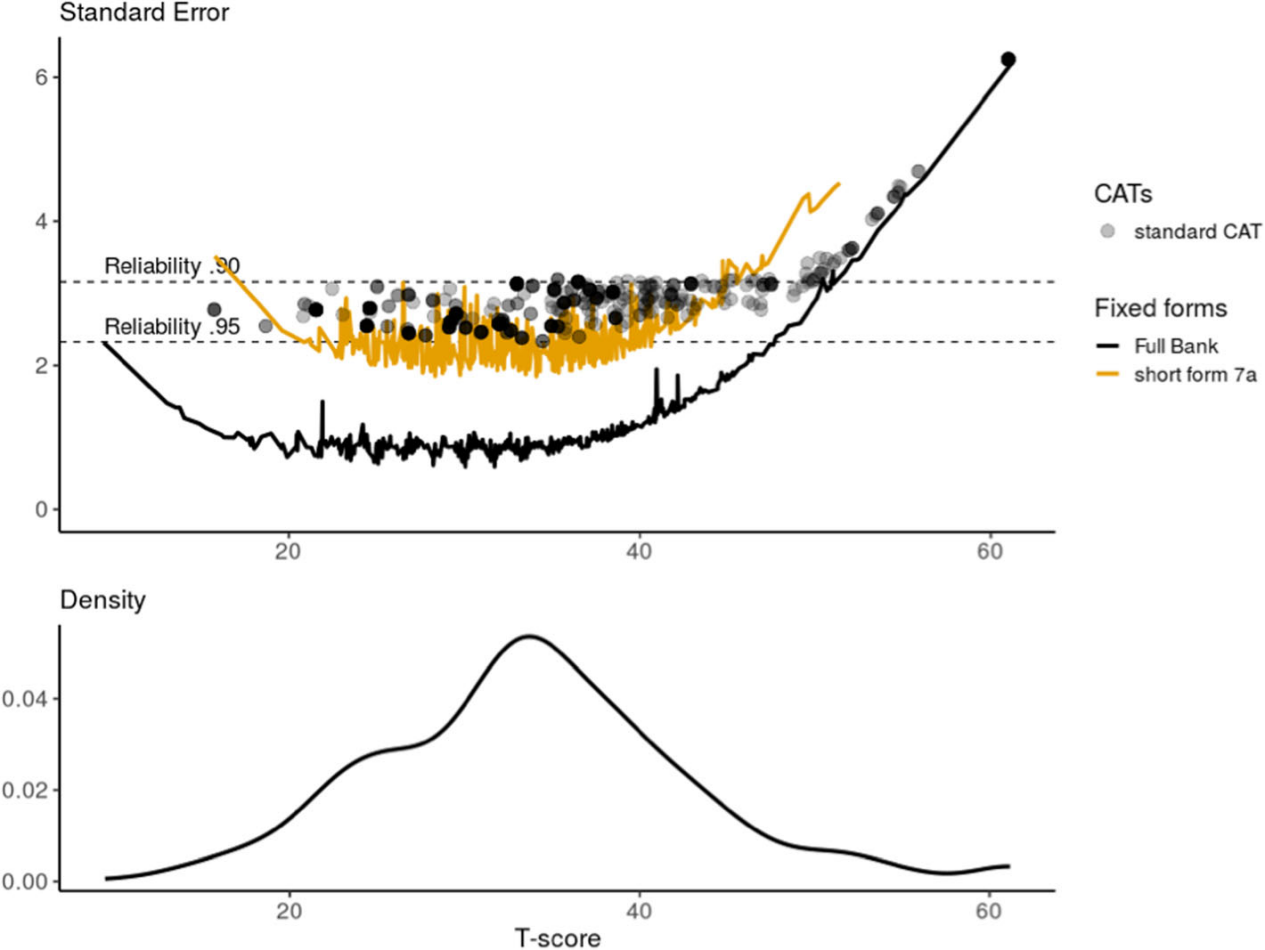
Reliability of the DF-PROMIS-UE v2.0 when using different applications (full item bank, 7-item short form and simulated standard CAT. Shading represents many of the same scores. The density plot represents the distribution of T-scores in the study sample

The three items with the highest information at *θ* = 0 (average of this Dutch sample) were PFB30 (*“Are you able to open a new milk carton?”),* PFA28 (*“Are you able to open a can with a hand can opener?”)* and PFA18 *(“Are you able to use a hammer to pound a nail?”).* Figure 1 shows the standard errors across T-scores for the full item bank, the standard 7-item short form and the two simulated CATs as well as the distribution of scores in the patient population based on the US item parameters. A theta could reliably be estimated (> 0.90) for 498/521 (95.6%) of the patients based on the full item bank and for all patients in the clinical range (T-score < 50). A theta could reliably be estimated for 460/521 (88.3%) of the patients based on the 7-item short form, and for all but five patients with T-scores lower than 45. Using the standard CAT, a reliability of > 0.90 was obtained for 469/515 (91.1%) of the patients and for all except three patients with a T-score < 50. The average number of items administered was 4.7 and 83.3% of the patients needed less than 7 items to get a reliable score. For the fixed 7-item CAT, a reliability of > 0.90 was obtained for 450/515 (87.4%) of the patients and for all patients with a T-score < 47.

#### Comparative precision

**Fig. 2:**
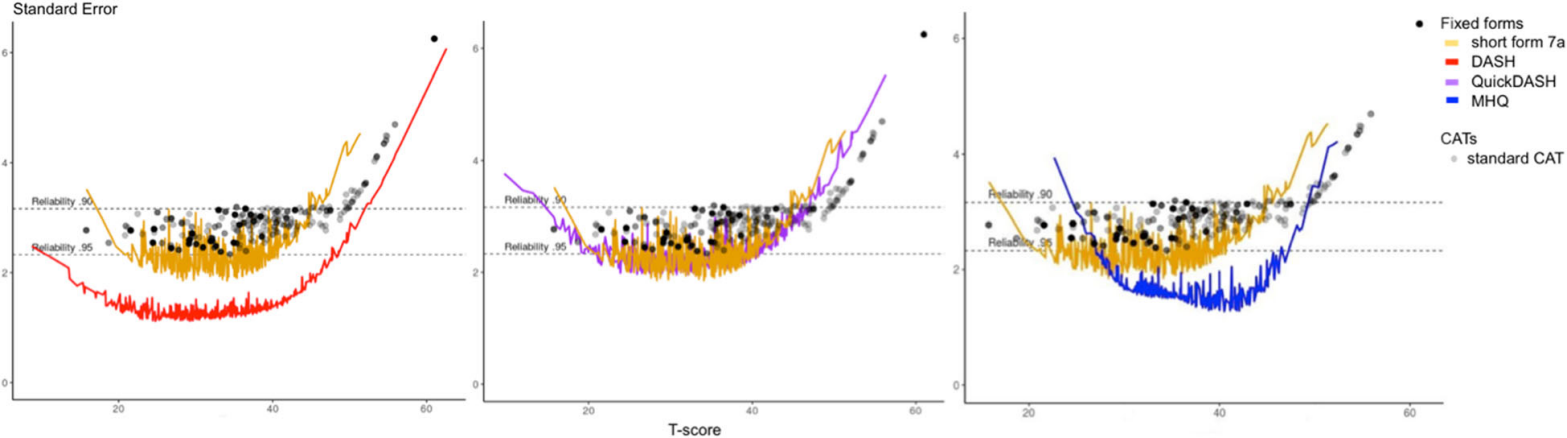
**a**-**c** Reliability of the CAT of the DF-PROMIS-UE v2.0 and the short form 7a, DASH, QuickDASH and MHQ

The DASH showed some lack of unidimensionality (CFI 0.91, TLI 0.90, RMSEA 0.13, SRMR 0.08) but all items fitted a GRM model. The QuickDASH also showed some lack of unidimensionality (CFI 0.94, TLI 0.92, RMSEA 0.15, SRMR 0.08) but all items fitted a GRM model. The MHQ-ADL showed better unidimensionality, although the RMSEA was higher than the criterion (CFI 0.99, TLI 0.99, RMSEA 0.13, SRMR 0.03) and all items fitted the GRM model. Figure 2 shows the reliability of the Dutch-Flemish DF-PROMIS-UE v2.0 short form and standard CAT versus the DASH, QuickDASH and MHQ-ADL. The 30-item DASH displayed better reliability than the DF-PROMIS-UE 7-item short form and standard CAT (Fig. 2a). The 11-item QuickDASH showed comparable reliability to the DF-PROMIS-UE CAT and short form (Fig. 2b). The 7-item MHQ-ADL displayed better reliability than the DF-PROMIS-UE 7-item short form and standard CAT for T-scores between T-scores of about 28 to 50, but for patients with low function the DF-PROMIS-UE v2.0 7-item short form and standard CAT performed better (Fig. 2c).

#### Relative efficiency

The DF-PROMIS-UE 7-item short form is on average more efficient than the full item bank. The DF-PROMIS-UE CAT is on average more efficient than the DF-PROMIS-UE full bank and 7-item short form and more efficient than the DASH, quickDASH and MHQ (Table 5). The DF-PROMIS-UE 7-item short form and full item bank are on average more efficient than the DASH and QuickDASH, but less efficient than the MHQ (Table 5).

**Table 5:** Mean relative efficiency of PROMIS measures versus legacy instruments

	DF-PROMIS-UE full bank (46 items)	DF-PROMIS-UE 7-item short form (7 items)	DF-PROMIS-UE standard CAT (average 4.7 items)
DF-PROMIS-UE full bank (46 items)		1.37	1.54
DF-PROMIS-UE 7-item short form (7 items)			1.30
DASH (30 items)	1.30	1.50	1.82
QuickDASH (11 items)	1.42	1.58	1.96
MHQ (7 items)	0.79	0.95	1.12

*DASH* Disability of Arm, Shoulder and Hand, *DF-PROMIS-UE v2.0* Dutch-Flemish translated version of the PROMIS Upper Extremity v2.0 item bank, *MHQ-ADL* Michigan Hand Questionnaire-Activities of Daily Living subscale

## Discussion

We validated the DF-PROMIS-UE v2.0 item bank in a Dutch population with upper extremity disorders. Together with two recent publications from our research group, this study provides the first complete foreign language validation of this item bank [16, 17]. Although we found some problems with the unidimensionality and the measurement invariance assumptions of the IRT model, a good IRT model fit and a high reliability across a wide range of the construct for the DF-PROMIS-UE v2.0 item bank were found.

With regard to unidimensionality, CFI and TLI values (0.93 and 0.93) were near the minimum criteria of 0.95, RMSEA was higher than the maximum criterion of 0.06 (0.10) and SRMR was slightly higher than the maximum criterion of 0.08 (0.09). A few studies reported on the validation of the PROMIS-UE v1.2 item bank, but none described the CFI, TLI, RMSEA, or SRMR values [39, 43–46]. A high RMSEA has been reported for many other PROMIS item banks [47–50]. It has been suggested that traditional cutoffs and standards for CFA fit statistics, are not suitable to establish unidimensionality of item banks measuring health concepts and bi-factor analysis has been suggested to examine whether a scale is sufficiently unidimensional [27, 51]. The bi-factor analysis results suggested sufficient unidimensionality of the DF-PROMIS-UE v2.0 item bank, which supports the use of IRT. However, a forced two-factor exploratory factor analyses showed that a 2-factor model, including one factor consisting of items referring to fine tactile function and one factor consisting of items referring to using the shoulder or lifting heavy objects could also be considered, although several items loaded on both factors.

Four item pairs had residual correlations above the critical value of 0.17. This local dependence is probably mainly due to redundancy-dependency (high degree of overlap within the content of the items), which we consider irrelevant to the measurement of upper extremity. However, items of pair PFA14r1 and PFA29r1 were both administered in the CAT in 14% of cases (in this order) and items of pair PFA36 and PFA44 were both administered in the CAT in 5.5% of cases (in this order). Moreover, PFA34 and PFA36 are both included in the standard short form 7a. Future studies should examine whether these results are consistent across studies and whether the CAT or short form would perform better if one ofor more of these local dependent items would be excluded. An additional 32 item pairs had negative residual correlations > − 0.20, suggesting multidimensionality. Most of these item pairs consisted of one item referring to fine tactile function and one item referring to using the shoulder or lifting heavy objects.

With respect to measurement invariance, we found no evidence for DIF due to age, but some items were flagged for DIF for gender, center, duration of complaints, and language. However, the impact of DIF on T-scores was considered negligible. Our study results therefore indicate that is legitimate to compare these groups when applying the DF-PROMIS-UE v2.0 item bank. However, for the items flagged for DIF regarding location of complaints, five out of the seven items included in the short form and five out of the nine items that were selected as one of the first three items in the CAT showed uniform DIF for location of complaints. Overall, the DIF results all seem to be related to a difference in performance between items regarding fine tactile function versus items regarding lifting heavy objects, which is in accordance with the forced two-factor EFA results. For example, all DIF results for location of complaint indicated that among patients with the same overall level of UE functioning, patients with only hand/wrist injuries indicated more problems with activities that involve fine tactile functioning and patients with only shoulder problems indicated more problems with activities involving heavy lifting tasks, reaching above shoulder level or behind the back. It is known that grip strength is merely a reflection of overall muscle strength and condition of a chain of muscles in the upper limb and at longterm follow-up is not severely impacted by hand or wrist injury [52–54]. In contrast, range of motion is significantly impacted by hand and wrist injuries and influences fine tactile functioning [53–55]. Therefore, we hypothesize that arm/shoulder problems impact heavy lifting activity, but to a lesser extent fine tactile functioning. In our previous study eight items were flagged DIF for language in the level 2 center patients [16], while in this study only four items were flagged for language DIF. This might be due to the slightly different study population of the level 1 center, including more patients with hand problems. More research in other populations with different distribution of injuries of the upper extremity should be performed to investigate possible multidimensionality and the impact of DIF for location of complaints on short form and CAT scores.

When studying measurement invariance for language (cross-cultural validity), we found 3 items with DIF. None of these DIF items are included in the standard 7a short form. Item PFM2 was selected as second item in the standard CAT in 15.9% of the patients, but the R^2^ change is small (0.0212) so the impact also should be small. Crins et al. examined language DIF of the PROMIS Physical Function v1.2 in a study in chronic pain patients. They found four items with language DIF, of which one item (PFB13 ‘*Are you able to carry a shopping bag or briefcase?’*) is also included in the PROMIS-UE v2.0 item bank. This item was not flagged for language DIF in our study. In contrast to our study, Crins et al. did not find DIF for any of the items flagged for DIF in our study that were also included in the PROMIS Physical Function v1.2 item bank [49]. It has been suggested that such differences can occur because most available DIF methods can detect whether there is DIF but cannot identify the exact DIF items due to parameter identification issues [56]. Our study and the study of Crins et al., found minimal impact of language DIF on T-scores, which suggests that the original US item parameters can be used for calculating the T-scores of the DF-PROMIS-UE v2.0 bank.

We found high reliability of simulated standard CAT T-scores with a reliability of > 0.90 (which has been considered a minimum requirement for use of PROMs in individual patients [57]) in 91.7% of the patients and in all patients within the clinical range, with on average only 4.7 items. The short form 7a had a reliability of > 0.90 in 88.5% of the patients. The short form was slightly more reliable than the standard CAT in the middle of the scale for T-scores between 18 and 45 but performed less than the CAT in patients with low function (range of T-score in the study population was 11–61). Both the standard CAT and the short form had sufficient reliability but the CAT required less items. The DASH displayed better reliability than the DF-PROMIS-UE v2.0 standard CAT and 7-item short form, while the QuickDASH displayed comparable reliability. However, the DASH requires 30 items, which may be considered too much for use in daily clinical practice. The MHQ-ADL is less reliable than the DF-PROMIS-UE v2.0 measures in patients with low functioning. Future studies should examine whether it is possible to further improve the standard CAT by choosing another starting item. Currently, item PFM16 is being used (*‘Are you able to pass a 20-pound (10 kg) turkey or ham to other people at the table?’*), but this item is less informative (ranked 14) in the Dutch sample and was flagged for language DIF in the level 2 traumacenter [16].

For adequate interpretation, a PROM has to be validated in the language in which it will be used, as we have done for the DF-PROMIS-UE v2.0. Van Eck et al. have performed validation of the DASH-Dutch Language Version and showed that it also measures a unidimensional trait [19]. Iordens et al. performed validation of the Dutch translated version of the QuickDASH [58]. Unfortunately, to our knowledge, the MHQ has not been validated in the Dutch language. This might hamper the interpretability of the outcome presented in this study with respect to the MHQ. On the other hand, our own study provides evidence for the adequate unidimensionality and reliability of the MHQ-ADL.

When reporting on outcomes of UE disorders in literature, extensive core sets including functional outcomes and PROMs have been suggested to improve comparability of studies [59, 60]. However, for clinical practice, a more practical ‘lean’ core set is advisable including a PROM with low burden for the patient and clinician. An advantage of the incorporating the DF-PROMIS-UE v2.0 in this ‘lean’ core set is that it has high correlation with other PROMs reporting on UE disorders, it decreases burden for patients and clinicians and it will allow clinicians to speak a ‘common language’ with regard to outcome reporting [61, 62]. However, the PROM should be able to detect clinical relevant change as expressed in the Minimal Important Change (MIC). De Vet et al. defined MIC as ‘the smallest change in construct to be measured which patients perceive as important’ [63]. The MIC threshold is very important in daily practice, where clinicians can compare at a patients’ individual level the current and previous values of outcome measures of interest. The MIC has been estimated for the DASH, QuickDASH, and MHQ [58, 64, 65]. However, for the PROMIS-UE v2.0 a MIC has not been established. Future research regarding test-retest reliability, smallest detectable change, and MICs is mandatory to be able to interpret outcome as reported with the DF-PROMIS-UE v2.0 in clinical practice.

## Conclusions

The DF-PROMIS-UE v2.0 item bank showed sufficient psychometric properties in a Dutch population with injuries of the upper extremity. This item bank is now ready for use as CAT in research and clinical practice and will be made available through the Dutch-Flemish Assessment Center (http://www.dutchflemishpromis.nl). However, more research on possible multidimensionality and impact of DIF for location of complaints on short form and CAT scores is recommended. Furthermore, test-retest reliability, responsiveness, and MICs need to be assessed in future studies. DF-PROMIS-UE v2.0 CATs allow reliable and valid measurement of outcome following musculoskeletal disorders of the upper extremity in an efficient and user-friendly way with limited administration time.

## Figures and Tables

**Fig. 3:**
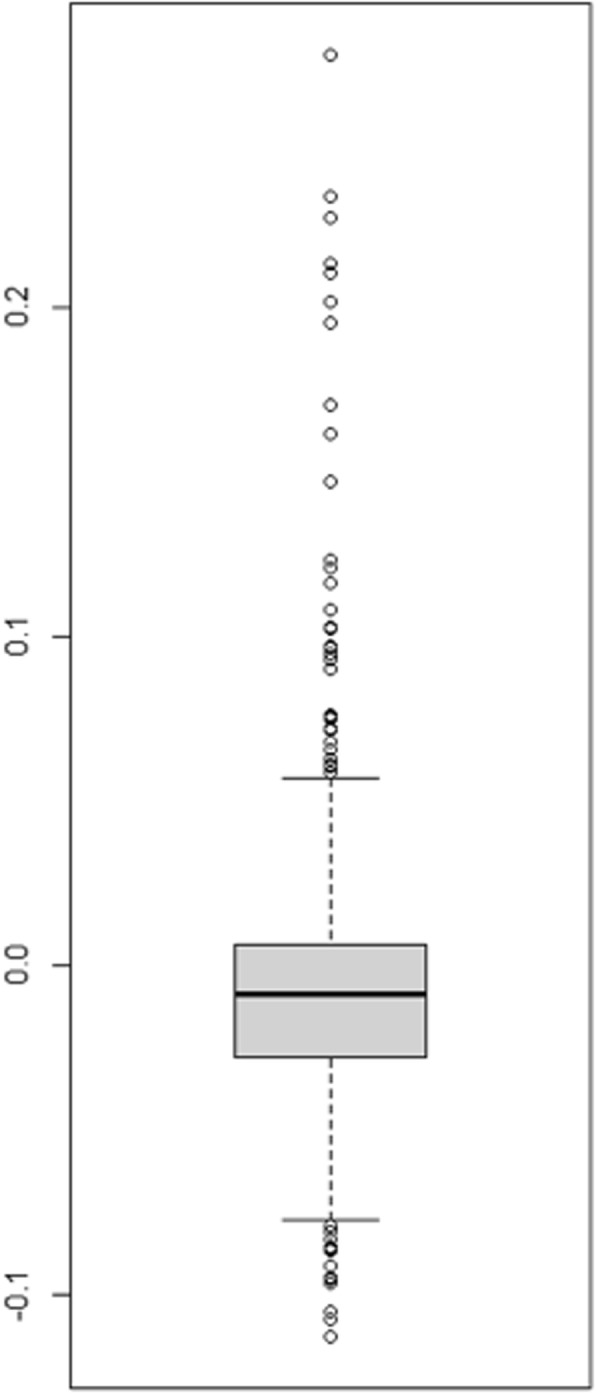
Differences between the initial theta and theta corrected for DIF for location of complaints. The box shows the interquartile range, representing the middle 50% of the differences

## Data Availability

The Dutch-Flemish dataset used and analyzed during the current study are available from the corresponding author on reasonable request. The US dataset is publicly available on the HealthMeasures Dataverse 10.7910/DVN/IHNIRH.

## Appendix 1

**Table 6:** Forced two-factor EFA

Item ID	Item stem	Rotated factor loadings	
		factor 1	factor 2
PFA14r1	Are you able to carry a heavy object (over 10 pounds/5 kg)?	0.275	0.724
PFA16r1	Are you able to dress yourself, including tying shoelaces and buttoning your clothes?	0.604	0.511
PFA17	Are you able to reach into a high cupboard?	0.208	0.791
PFA18	Are you able to use a hammer to pound a nail?	0.583	0.541
PFA20	Are you able to cut your food using eating utensils?	0.802	0.300
PFA28	Are you able to open a can with a hand can opener?	0.706	0.442
PFA29r1	Are you able to pull heavy objects (10 pounds/5 kg) towards yourself?	0.383	0.734
PFA34	Are you able to wash your back?	0.340	0.700
PFA35	Are you able to open and close a zipper?	0.704	0.331
PFA36	Are you able to put on and take off a coat or jacket?	0.444	0.574
PFA38	Are you able to dry your back with a towel?	0.486	0.615
PFA40	Are you able to turn a key in a lock?	0.683	0.330
PFA43r1	Are you able to write with a pen or pencil?	0.738	0.235
PFA44	Are you able to put on a shirt or blouse?	0.541	0.516
PFA48	Are you able to peel fruit?	0.828	0.250
PFA50	Are you able to brush your teeth?	0.646	0.304
PFA54	Are you able to button your shirt?	0.781	0.287
PFB11	Are you able to wash dishes, pots, and utensils by hand while standing at a sink?	0.747	0.356
PFB13	Are you able to carry a shopping bag or briefcase?	0.405	0.660
PFB15r1	Are you able to change the bulb in a table lamp?	0.696	0.409
PFB16r1	Are you able to press with your index finger (for example ringing a doorbell)?	0.582	0.271
PFB18	Are you able to shave your face or apply makeup?	0.709	0.380
PFB19r1	Are you able to squeeze a new tube of toothpaste?	0.788	0.271
PFB20r1	Are you able to cut a piece of paper with scissors?	0.823	0.243
PFB21r1	Are you able to pick up coins from a table top?	0.732	0.169
PFB22	Are you able to hold a plate full of food?	0.667	0.476
PFB23r1	Are you able to poor liquid from a bottle into a glass?	0.675	0.449
PFB25	Are you able to push open a door after turning the knob?	0.548	0.469
PFB26	Are you able to shampoo your hair?	0.617	0.514
PFB27	Are you able to tie a knot or a bow?	0.813	0.280
PFB28r1	Are you able to lift 10 pounds (5 kg) above your shoulder?	0.133	0.870
PFB30	Are you able to open a new milk carton?	0.777	0.383
PFB31r1	Are you able to open car doors?	0.639	0.475
PFB33	Are you able to remove something from your back pocket?	0.484	0.544
PFB34	Are you able to change a light bulb overhead?	0.361	0.744
PFB36	Are you able to put on a pullover sweater?	0.413	0.645
PFB37r1	Are you able to reach and get down a 5 pound (2 kg) object from above your head?	0.722	0.353
PFB39r1	Are you able to reach and get down a 5 pound (2 kg) object from above your head?	0.288	0.830
PFB41	Are you able to trim your fingernails?	0.754	0.245
PFB56r1	Are you able to lift one pound (0.5 kg) to shoulder level without bending your elbow?	0.274	0.740
PFC43	Are you able to use your hands, suchs as for turning faucets, using kitchen gadgets, or sewing?	0.769	0.278
PFC49	Are you able to water a house plant?	0.639	0.424
PFM2	Are you able to lift a heavy painting or picture to hang on your wall above eye-level?	0.369	0.749
PFM16	Are you able to pass a 20-pound (10 kg) turkey or ham to other people at the table?	0.305	0.756
PFM18	Are you able to continuously swing a baseball bat or tennis racket back and forth for 5 min?	0.236	0.765
PFC8	Does your health now limit you in opening a previously opened jar?	0.620	0.457

## Abbreviations

CAT: Computerized Adaptive Testing
CFA: Confirmatory factor analysis
CFI: Comparative Fit Index
DASH: Disability of Arm, Shoulder and Hand
DF-PROMIS-UE v2.0: Dutch-Flemish translation of the Patient-Reported Outcomes Measurement Information System Physical Functioning – Upper Extremity version 2.0
DIF: Differential Item Functioning
EAP: *Expected a posteriori*
ECV: Explained Common Variance
EFA: Exploratory Factor Analysis
GRM: Graded Response Model
IRT: Item Response Theory
MHQ-ADL: Michigan Hand Questionnaire subscale Activities of Daily Living
PROM: Patient Reported Outcome Measure
PROMIS: Patient-Reported Outcomes Measurement Information System
PRWE: Patient Reported Wrist Evaluation
RMSEA: Root Means Square Error of Approximation
SD: Standard deviation
SE: Standard error
SRMR: Standardized Root Mean Residual
TLI: Tucker-Lewis Index
UE: Upper extremity
US: United States
WLSMV: Weighted Least Squares with Mean and Variance adjustment
